# Adaptive Initialization Method for K-Means Algorithm

**DOI:** 10.3389/frai.2021.740817

**Published:** 2021-11-25

**Authors:** Jie Yang, Yu-Kai Wang, Xin Yao, Chin-Teng Lin

**Affiliations:** ^1^ Computational Intelligence and Brain Computer Interface Lab, Australian Artificial Intelligence Institute, FEIT, University of Technology Sydney, Sydney, NSW, Australia; ^2^ Shenzhen Key Laboratory of Computational Intelligence, Department of Computer Science and Engineering, Southern University of Science and Technology, Shenzhen, China; ^3^ CERCIA, School of Computer Science, University of Birmingham, Birmingham, United Kingdom

**Keywords:** k-means, adaptive, initialization method, initial cluster centers, clustering

## Abstract

The K-means algorithm is a widely used clustering algorithm that offers simplicity and efficiency. However, the traditional K-means algorithm uses a random method to determine the initial cluster centers, which make clustering results prone to local optima and then result in worse clustering performance. In this research, we propose an adaptive initialization method for the K-means algorithm (AIMK) which can adapt to the various characteristics in different datasets and obtain better clustering performance with stable results. For larger or higher-dimensional datasets, we even leverage random sampling in AIMK (name as AIMK-RS) to reduce the time complexity. 22 real-world datasets were applied for performance comparisons. The experimental results show AIMK and AIMK-RS outperform the current initialization methods and several well-known clustering algorithms. Specifically, AIMK-RS can significantly reduce the time complexity to O (*n*). Moreover, we exploit AIMK to initialize K-medoids and spectral clustering, and better performance is also explored. The above results demonstrate superior performance and good scalability by AIMK or AIMK-RS. In the future, we would like to apply AIMK to more partition-based clustering algorithms to solve real-life practical problems.

## Introduction

The clustering algorithm is a classical algorithm in the field of data mining. It is used in virtually all natural and social sciences and has played a central role in various fields such as biology, astronomy, psychology, medicine, and chemistry (Shah and Koltun 2017). For example, in the commercial field, Horng-Jinh Chang et al. proposed an anticipation model of potential customers’ purchasing behavior based on clustering analysis (Chang et al., 2007). In the biology field, clustering is of central importance for the analysis of genetic data, as it is used to identify putative cell types (Kiselev et al., 2019). In addition, the applications of the clustering algorithm also include image segmentation, object or character recognition (Dorai and Jain 1995), (Connell and Jain 1998) and data reduction (Huang 1997) (Jiang et al., 2014). The clustering algorithm mainly includes hierarchy-based algorithms, partition-based algorithms, density-based algorithms, model-based algorithms and grid-based algorithms (Saxena et al., 2017).

The K-means algorithm is widely used because of its simplicity and efficiency (MacQueen 1967). Even it was proposed for more than 50 years, there are still many related studies today (Bu et al., 2019; Lai et al., 2019; I.; Khan et al., 2019). The K-means algorithm is a classic partition-based clustering algorithm. However, the traditional K-means algorithm uses the random method to determine the initial cluster centers, which make clustering results prone to local optima and then result in worse clustering performance. To overcome this disadvantage, many improved methods have been proposed. However, providing an optimal partition is an NP hard problem under a specific metric (Redmond and Heneghan, 2007).

Forgy randomly selected K points from the data as the initial cluster centers without a theoretical basis, and the final clustering results more easily fell into a local optimum (Forgy, 1965). Jancey’s method assigned a randomly generated synthetic point from the data space to each initial clustering center (Jancey 1966). However, some of these centers may be quite distant from any of the points, which might lead to the formation of empty clusters. MacQueen proposed using the first K points in the dataset as the initial centers. The disadvantage of this approach is that the method is extremely sensitive to data order (MacQueen 1967). In addition, the above methods do not take into account the characteristics of data distribution, using randomly generated points or synthetic points as the initial cluster centers, resulting in poor and unstable clustering results (Yang et al., 2017a). Selecting clustering centers is actually selecting the representative points for specific classes. The density of data points can be used to measure the representativeness of points. Redmond et al. estimated the density distribution of the dataset by constructing a Kd-tree (Redmond and Heneghan 2007), but its density calculation method was unreasonable (Wang et al., 2009). Zhang et al. proposed an initialization method based on density Canopy with complexity O(n2) (Zhang et al., 2018). In addition, Cao et al. used the neighborhood-based rough set mode to measure the representativeness of the points to generate the initial cluster centers, but the method was sensitive to parameters (Cao et al., 2009). Khan et al. calculated the representative points from the dimensions of the data points based on the principle of data compression (S. S. Khan and Ahmad 2004). The overall effect of this method is good, but its complexity is positively related to the dimensionality of the data and is not applicable to high-dimensional data. Based on the minimum spanning tree (MST), Yang et al. selected representative points, which are also called skeleton points, from the datasets and then regarded some skeleton points that are far away from each other as the final initial cluster centers (Yang et al., 2017b). However, the complexity of this method is quadratic. S. Manochandar et al. chose representative points by computing the eigenvectors of the dataset-relative matrix, but this method has only been proven to reduce the sum of squared error (SSE) of the partitions, instead of to improve objective clustering accuracy (Manochandar et al., 2020). In addition to the density of the data points, the distance between the data points is also regarded as one of the criteria for selecting the initial cluster centers. Gonzalez proposed a maximin method; the idea is to select the data points, which are as far as possible from each other, as the initial cluster centers, to make the cluster centers more evenly dispersed in each class (Gonzalez 1985). However, this method has strong randomness, resulting in unstable clustering results. Arthur et al. proposed K-means++ (Arthur and Vassilvitskii 2007), which has disadvantages similar to the maximin method. For example, K-means++ will result in unstable clustering results because of the randomly selected first cluster center, or it may generate no representative initial cluster centers. Murugesan et al. determined the initial cluster centers by the maximum average distance model, but this model is sensitive to outliers (Murugesan and Punniyamoorthy, 2020). To obtain better clustering results, some methods consider both the representativeness of data points and the distance between data points. Rodriguez et al. proposed a new clustering algorithm based on density peaks and proposed a method to generate cluster centers based on both density and distance (Rodriguez and Laio 2014). However, none of the above-mentioned methods can dynamically adapt to datasets with various characteristics (Yang et al., 2017a).

Yang et al. proposed a K-means initialization method based on a hybrid distance, which can dynamically adapt to datasets with various characteristics (Yang et al., 2017a). The method considers both the density and the distance and uses a parameter to adjust the proportion of the two factors. They also proposed an internal clustering validation index, named the clustering validation index based on the neighbors (CVN), to select the optimal clustering results. However, this method also has shortcomings, such as 1) when calculating density, the threshold cannot be uniquely determined, resulting in unstable results. 2) Heavily depending on adjusting the parameter, the parameter must be adjusted five times to obtain better clustering results. 3) In some cases, the CVN index values calculated using different parameter settings are equal. At this time, CVN cannot be used to select better clustering results. 4) The time complexity of the algorithm is O ( $n^{2}$ ), which is difficult to apply to large datasets.

In this paper, we propose an adaptive initialization method for the K-means algorithm (AIMK), which not only adapts to datasets with various characteristics but also requires only two runs to obtain better clustering results. Also, we propose the AIMK-RS based on random sampling to reduce the time complexity of the AIMK to O(*n*). AIMK-RS is easily applied to large and high-dimensional datasets. First, we propose a new threshold to calculate the density of the data points based on the skeleton points of MST. Second, we compute the hybrid distances based on the density of the data points. Finally, we select K data points, where the hybrid distances among them are relatively large, as the final cluster centers. In addition, we apply random sampling to AIMK to obtain the AIMK-RS, whose time complexity is only O(*n*). We also exploit AIMK to initialize the variants of K-means, such as K-medoids and spectral clustering. And it can still obtain better clustering performance, which proves that AIMK also has good scalability. This paper is organized as follows. In the Adaptive Initialization Method section, an adaptive initialization method for K-means is presented. In the Experiments and Results section, the experimental studies are presented and discussed. Finally, in the Conclusion section, the relevant conclusions are drawn.

The following are the main contributions of this paper:

1) Proposing an adaptive initialization method for the K-means algorithm (AIMK), which not only adapts to datasets with various characteristics but also requires only two runs to obtain better clustering results;
2) Proposing the AIMK-RS based on random sampling to reduce the time complexity of the AIMK to O(*n*);
3) Proposing a new threshold to estimate the density of the data points based on the skeleton points of MST;
4) Exploiting AIMK to initialize the variants of K-means, such as K-medoids and spectral clustering to prove AIMK’s good scalability;
5) Comprehensive experiments tested on 22 real-world datasets validate the superiority of the proposed methods compared with the 11 current state-of-the-art methods.

## Adaptive Initialization Method for the K-Means Algorithm

In this section, we describe the algorithm for selecting the initial cluster centers in detail. First, several concepts involving this algorithm are presented.

### Skeleton Points

In a previous study, Jie et al. proposed a new compressed representation, named skeleton points, from the original datasets based on an MST and regarded them as candidates for cluster centers (Yang et al., 2017b). In contrast, we leverage the skeleton points to determine the threshold for calculating the density of data points because they can reflect the characteristics of the datasets to some extent. In the beginning, we introduce how to construct an MST using the original dataset.

Let *X* denote a dataset with *K* clusters and *n* data points: $X=\left\{x_{i}|x_{i}\in R^{p},i=1,2,\ldots ,n\right\}$ . To use the MST to get the skeleton points, dataset *X* should be represented by the undirected complete weighted graph G=(V,E) , where $V=\left\{v_{1},v_{2},\ldots ,v_{n}\right\}$ ., $\;\left|E\right|=\frac{n\left(n-1\right)}{2}$ . Each data point $x_{i}$ in dataset *X* corresponds to a vertex $v_{i}\in V$ in graph *G*, and the data point $x_{i}\;$ (*i* = 1, 2,…, *n*) and vertex $v_{i}$ (*i* = 1, 2,…, *n*) have a one-to-one correlation. The number of vertices in graph *G* is the same as the number of data points $x_{i}$ in dataset *X*. The distance between any two vertices is equal to the edge weights between that two data points.

The Prim algorithm (Prim 1957) can be used to generate the MST of *G*, which can be described as follows:

Step 1: Pick any vertex $v_{i}$ from graph *G* to be the root of the tree.
Step 2: Grow the tree by one edge: of the edges that connect the tree to vertices not yet in the tree, find the minimum-weight edge from *G* and transfer it to the tree.
Step 3: Repeat Step 2 (until the tree contains all vertices in graph *G*).

We create an MST from the original dataset using the procedures above, and then show how to extract skeleton points from the MST. We start by introducing a concept called the number of adjacent data points, and then we use it to generate skeleton points.

Let $T=\left(V,E_{T}\right)$ be a minimum spanning tree of G=(V,E) , where $V=\left\{v_{1},v_{2},\ldots ,v_{n}\right\}$ , $\;E_{T}=\left\{e_{1},e_{2},\ldots ,e_{n-1}\right\}$ , $e_{i}\in E\left(G\right)$ .

Definition 2.1: (Number of adjacent data points, Yang et al., 2017b) Let $U_{i}$ be the set of vertices of *T* with a degree *i* and $W_{i}$ be the complementary set of $U_{i}$ , that is, $W_{i}=V\backslash U_{i}$ . For $U_{i}$ , the number of adjacent data points, denoted as $f_{i}$ , is the number of vertex in $W_{i}$ being adjacent to vertex in $U_{i}$ .

Note that only add 1 to $f_{i}$ under the circumstance of one vertex in $W_{i}$ being adjacent to more than one vertex in $U_{i}$ .

Theorem 2.1: If anyone vertex in $W_{i}$ is adjacent to one and only one vertex in $U_{1}$ , then $f_{1}=\left|U_{1}\right|$ , otherwise $f_{1}<\left|U_{1}\right|$ .

Now, we introduce how to leverage the number of adjacent data points $f_{i}$ to obtain the skeleton points.

Definition 2.2: (Skeleton Point, Yang et al., 2017b) Suppose the maximum degree of *T* be *m*; then, $V=U_{1}\cup U_{2}\cup \ldots \cup U_{m}$ . Let $F=\arg\underset{i}{\max}f_{i}$ . The skeleton points, denoted as *S*, are the vertices of *T* with the degree being greater than or equal to *F*. Therefore, $S=U_{F}\cup U_{F+1}\cup \ldots \cup U_{m}$ .

**FIGURE 1:**
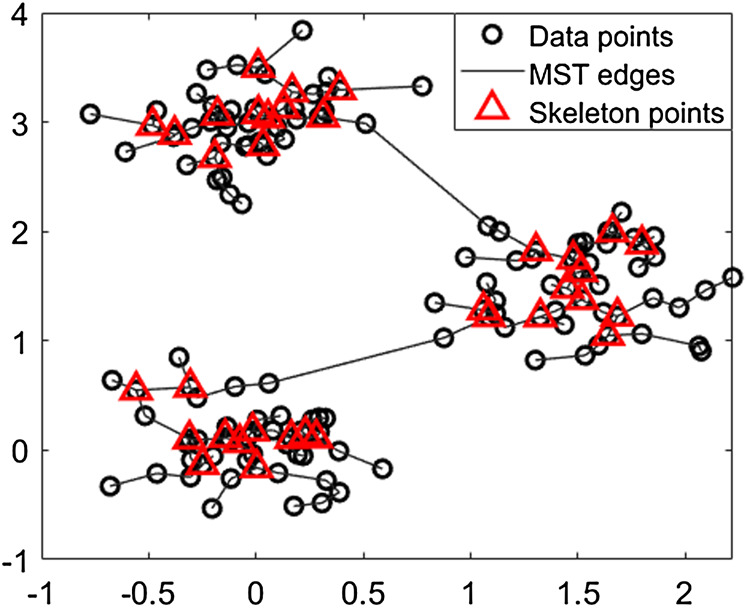
We generate a synthetic dataset, then construct the MST and calculat the skeleton points according to Definitions 2.1–2.2; they are enclosed by the triangles. As shown, the skeleton points are a type of compressed representation based on the characteristic of the dataset.

We generate a synthetic dataset, then construct the MST and calculate the skeleton points according to the Definitions 2.1–2.2 which are enclosed by the triangles, as shown in Figure 1. Next, we introduce the threshold for calculating the density of data points.

### Threshold

Definition 2.3: (Threshold) In $T=\left(V,E_{T}\right)$ , suppose the number of skeleton points S is *s*; if the maximum weights of adjacent edges of each skeleton point can be denoted as $\left\{w_{1},w_{2},\ldots ,w_{s}\right\}$ , then we define a threshold as

$$Thr=\frac{\sum_{i=1}^{s}w_{i}}{s}$$ (1)

In an MST, the adjacent edge weights of vertices can reflect the distribution characteristics of the area where the vertices are located. While vertices contain a large number of unimportant points or outliers, we only focus on the skeleton points. In summary, when calculating the threshold, we only consider the adjacent edge weights of the skeleton points, and the mean value of the maximum weights of adjacent edges of each skeleton point is taken as the threshold.

### Density of Vertices

In the following section, we introduce how to calculate the density of data points using the threshold *Thr.* We first construct a *Thr*-Connected Graph (*TCG*).

Definition 2.4: (*Thr*-Connected Graph) In dataset *X*, if $\text{d}\left(x_{i},x_{j}\right)\leq Thr$ , then add an edge between data points $x_{i}$ and $x_{j}$ ; this is called a *Thr*-Connected Graph (*TCG*), where $d\left(x_{i},x_{j}\right)$ is the distance between the data point $x_{i}$ and $x_{j}$ . Each data point $x_{i}$ in dataset *X* corresponds to a vertex $v_{i}\in V$ in graph *TCG*.

Definition 2.5: (density of $v_{i}$ ) In *TCG*, the mean distance between the vertex $v_{i}$ and the vertices connected to vertex $v_{i}$ , denoted as $v_{j}$ , is

$$D\left(v_{i}\right)=\frac{1}{k}\underset{v_{i,j}\in V}{\sum }d\left(v_{i},v_{j}\right)$$ (2)

where *k* is the number of vertices $v_{j}$ .

Suppose $v^{k}=\left\{v_{i}|\;the\;number\;of\;vertices\text{ connected }to\;vertex\;v_{i}\;is\;k\right\}$ , $\;D_{max}^{k}=\underset{v_{i}\in v^{k}}{\max}D\left(v_{i}\right)$ , and $\;D_{min}^{k}=\underset{v_{i}\in v^{k}}{\min}D\left(v_{i}\right)$ ; then, the density of $v_{i}$ is

$$\rho_{i}=\{\begin{array}{c} 0,\;\;\;\;\;k=0\;\;\;\;\;\;\;\;\;\\ k+\frac{D_{max}^{k}-D\left(v_{i}\right)}{D_{max}^{k}-D_{min}^{k}+\text{ε}},\;k\neq 0 \end{array}$$ (3)

To make $\frac{D_{max}^{k}-D\left(v_{i}\right)}{D_{max}^{k}-D_{min}^{k}}$ <1, we add an infinite decimal *ε* to its denominator, where ε→0+ .

### Hybrid Distance

If the distance among the initial cluster centers is small, it is easy to make the K-means algorithm fall into a local optimum. However, if only the distance factor is considered, it is possible to use the outlier as the initial cluster center. Jie. et al. proposed a new distance, a hybrid distance, to solve this problem (Yang et al., 2017a). Hybrid distance considers the distance and density of the cluster centers at the same time so that the selected cluster centers are far away from each other and have a higher density.

Definition 2.6: (Hybrid distance between $v_{i}$ ) In *TCG*, suppose $d_{max}=\underset{1\leq i,j\leq n,i\neq j}{\max}\left(d\left(v_{i},v_{j}\right)\right)$ , $d_{min}=\underset{1\leq i,j\leq n,i\neq j}{\min}\left(d\left(v_{i},v_{j}\right)\right)$ , $P_{max}=\underset{1\leq i,j\leq n,i\neq j}{\max}\left(\rho_{i}+\rho_{j}\right)$ , and $\;P_{min}=\underset{1\leq i,j\leq n,i\neq j}{\min}\left(\rho_{i}+\rho_{j}\right)$ ; the hybrid distance between the vertex $v_{i}$ and $v_{j}$ is

$$H\left(v_{i},v_{j}\right)=\lambda {\left[\frac{d\left(v_{i},v_{j}\right)-d_{min}}{d_{max}-d_{min}}\right]}^{2}+\left(1-\lambda \right){\left[\frac{\left(\rho_{i}+\rho_{j}\right)-P_{min}}{P_{max}-P_{min}}\right]}^{2}$$ (4)

where λ is a hyperparameter, normally set by 0 or 1 in practice; this is explained in detail in the following section.

### The Algorithm of the Proposed AIMK

Now we present the algorithm for determining the initial cluster centers based on the above-defined concepts. The details are as follows:

Algorithm 1 Algorithm of the proposed AIMK.

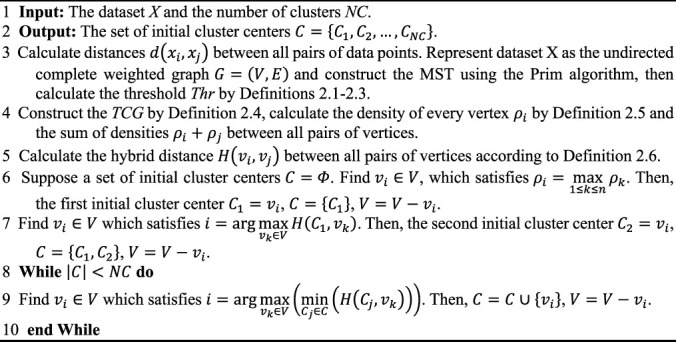

### Algorithm Analysis

Clustering is NP-hard. No published optimization method provides theoretical guarantees for optimal partition of K-means for all datasets, even if the number of clusters is fixed to 2 (Dasgupta 2008). Due to the intractability of NP-hard problems, clustering algorithms are evaluated in terms of empirical performance on standard datasets. Therefore, in previous studies, many heuristic clustering algorithms have been proposed, one of the most well-known is clustering by fast search and find of density peaks (SFDP) (Rodriguez and Laio 2014). SFDP determines the cluster centers by measuring the two factors, the Gaussian kernel density of data points and the density-relative distance between data points. These two factors inspired the research of this paper. The proposed model is based on the skeleton points in the MST to estimate the density of each data point, and then combines the dissimilarity (i.e., distance) between the data points to calculate the hybrid distance matrix, and finally selects K data points, where the hybrid distances among them are relatively large, as the final cluster centers. In the experiment part, lots of test cases have demonstrated the effectiveness of the proposed model.

### Time Complexity Analysis

According to the Algorithm 1, the time complexity of AIMK is analyzed as follows. In Step 3, the time complexity of computation of the distance between all pairs of vertices and the Prim algorithm is $\text{O}\left(n^{2}\right)$ , and the time complexity of calculation of the threshold *Thr* is O(n) . Construction of the *TCG* and calculation of the density of every vertex $\rho_{i}$ requires O(n) in Step 4, and the computation of the sum of densities $\rho_{i}+\rho_{j}$ between all pairs of vertices requires O(n) . In Step 5, because the distance and the sum of densities between all pairs of vertices have been obtained in Step 3 and Step 4, the time complexity of the calculation of the hybrid distance $H\left(v_{i},v_{j}\right)$ between all pairs of vertices is O(n) . Determination of the first and second initial cluster centers requires O(n) in Step 6 and Step 7. In Steps 8–10, the remaining initial cluster centers are selected, in which the time complexity is less than NC∗n and approximately equal to O(NC∗n) *.* Because normally the number of clusters NC≪n , the entire time complexity of AIMK is $\text{O}\left(n^{2}\right)$ .

### Reducing Complexity of AIMK by Sampling (AIMK-RS)

Due to the time complexity $O\left(n^{2}\right)$ , it is difficult to apply AIMK to large or high-dimensional datasets. To solve this problem, we consider random sampling to extract $\sqrt{n}$ samples from the original dataset, where n means the number of samples of the dataset, and then use these samples as the input for AIMK. It is worth mentioning that to make the $\sqrt{n}$ samples fully express the characteristics of the original dataset, we recommend using random sampling to reduce complexity only when the number of clusters K≪n . In this way, the time complexity of AIMK will be reduced to O(n) . AIMK after random sampling, is denoted as AIMK-RS. The time complexities of all baselines (will be introduced in experiment part), AIMK and AIMK-RS are listed in Table 1.

**TABLE 1:** Comparison of Time complexity.

Algorithm	Time Complexity
K-means	O(n)
K-means++	O(n)
KT	O(nlogn)
MSTI	$O\left(n^{2}\right)$
HD	$O\left(n^{2}\right)$
K-medoids	$O\left(n^{2}\right)$
SFDP	$O\left(n^{2}\right)$
FCM	O(n)
Sing-linkage	$O\left(n^{2}logn\right)$
Self-tuning Spectral	$O\left(n^{3}\right)$
FINCH	O(nlogn)
AIMK	$O\left(n^{2}\right)$
AIMK-RS	O(n)

## Experiments

In this section, we mainly introduce the public datasets required for the experiment, well-known clustering algorithms, and several validation indices to evaluate the overall clustering performance and comparisons.

### Datasets

In experiments, we use 22 real-world datasets (16 normal and six larger or higher-dimensional from the UCI Machine Learning Respository (https://archive.ics.uci.edu/ml/datasets) and LIBSVM official website (https://www.csie.ntu.edu.tw/∼cjlin/libsvmtools/datasets). The datasets include Breast-cancer, Shuttle, Pendigits, Colon-cancer, Zoo, Haberman, Svmguide2, Wine, Ionosphere, Leukemia, Gisette, Splice, Svmguide4, Liver-disorders, Soybean-small, Balance-scale, Ijcnn1, Phishing, Protein, Mushrooms, SensIT Vehicle (seismic), SensIT Vehicle (combined). The description of the datasets is as shown in Table 2.

**TABLE 2:** Description of the 22 datasets.

Dataset	Number of Instances	Number of Attributes	Number of Classes
Breast-cancer	683	10	2
Shuttle	14,500	9	7
Pendigits	3,498	16	10
Colon-cancer	62	2000	2
Zoo	101	16	7
Haberman	306	3	2
Svmguide2	391	20	3
Wine	178	13	3
Ionosphere	351	34	2
Leukemia	34	7,129	2
Gisette	1,000	5,000	2
Splice	2,175	60	2
Svmguide4	312	10	6
Liver-disorders	200	5	2
Soybean-small	47	35	4
Balance-scale	625	4	3
Ijcnn1	91,701	22	2
Phishing	11,055	68	2
Protein	6,621	357	3
Mushrooms	8,124	112	2
SensIT Vehicle (seismic)	19,705	50	3
SensIT Vehicle (combined)	19,705	100	3

### State-of-The-Art Clustering Algorithms for Comparisons

We compare the clustering performance between AIMK and 11 selected clustering algorithms. For the sake of fairness, these baselines not only include initialization methods for K-means, such as K-means, K-means++, the method initializing K-means using kd-trees (KT) (Redmond and Heneghan 2007), the MST-based initializing K-means (MSTI) (Yang et al., 2017b), and the initialization method based on hybrid distance for K-means (HD) (Yang et al., 2017a), but also include some well-known clustering algorithms, such as K-medoids (Kaufman and Rousseeuw 2009), clustering by fast search and find of density peaks (SFDP) (Rodriguez and Laio 2014), fuzzy C-means clustering (FCM) (Bezdek et al., 1984), single-linkage hierarchical clustering (SH) (Johnson 1967), and self-tuning spectral clustering (SS) (Zelnik-Manor and Perona 2005), efficient parameter-free clustering using first neighbor relations (FINCH) (Sarfraz et al., 2019). Besides, since the results of K-means, K-means++, K-medoids, FCM, and SS are not unique, we take the average performance of 10 runs as the real performance. SFDP has a hyperparameter *dc*, ranging from 1–2% (Rodriguez and Laio 2014). We take the average performance while the hyperparameter equals 1, 1.1, 1.2, 1.3, 1.4, 1.5, 1.6, 1.7, 1.8, 1.9, and 2% because of the sensitivity of the hyperparameter. Furthermore, we take the ground-truth number of clusters as prior knowledge to choose the cluster centers in SFDP. For FINCH, we exploit the required number of clusters mode to ensure fairness of comparison. Note that all the baselines and AIMK used Euclidean distance as a metric. All experiments were performed in MatLab 2019b environment, and were conducted on a laptop with the 4-core Intel i7-10510U CPU clocked at 1.8 and 2.3 GHz and 16 GB memory.

### Validation Indices

To evaluate the performance of clustering algorithms, we exploit three widely used external clustering validation indices including Accuracy (ACC), Rand Index (RI), and F-measure. These indices are defined as follows:

$$\text{ACC}=\frac{\sum_{i=1}^{NC}P_{i}}{n}$$ (5)

$$\text{RI}=\frac{TP+TN}{TP+FP+FN+TN}$$ (6)

$$\text{Precision}=\frac{TP}{TP+FP}$$ (7)

$$\text{Recall}=\frac{TP}{TP+FN}$$ (8)

$$\text{F}-\text{measure}=\frac{2\text{∗}Precision\text{∗}Recall}{Precision+Recall}$$ (9)

where *n* denotes the number of objects. *NC* is the number of clusters. *P*_*i*_ is the number of objects that are correctly assigned. *TP* means true positive, *FP* means false positive, *FN* means false negative, and *TN* means true negative (Powers 2011).

## Results

In this section, we analyze the parameter setting of AIMK and then compare the proposed AIMK algorithm with other well-known clustering approaches. We then compare AIMK-RS with the two baselines with linear complexity on larger or higher-dimensional datasets. In addition, the AIMK is also applied to the variants of the K-means algorithm, K-medoids, and spectral clustering to prove the scalability.

### Sensitivity and Setting of λ

To analyze the sensitivity of the parameter λ, we run AIMK on 16 datasets: Breast-cancer, Shuttle, Pendigits, Colon-cancer, Zoo, Haberman, Svmguide2, Wine, Ionosphere, Leukemia, Gisette, Splice, Svmguide4, Liver-disorders, Soybean-small, and Balance-scale while λ is set as 0, 0.25, 0.5, 0.75, 1. Then, we use ACC, RI, and F-measure to evaluate the performance of AIMK on each dataset. The results are listed in Tables 3–5. The optimal results for the corresponding index are denoted in bold. As the results show, when λ is set as 0 or 1, the optimal results in each validation index can be always obtained in each dataset. This is because of K-means’ own iterative mechanism. Even though different initial cluster centers are obtained because of different settings of the parameter λ, the same clustering results are finally obtained after iterating. The HD algorithm is required to run five times to obtain a better clustering result (Yang et al., 2017a), but AIMK can obtain a better result with only two runs, that is when λ is set as 0 or 1, respectively. Therefore, in subsequent experiments, we only consider the results of AIMK when λ equals 0 or 1.

**TABLE 3:** AIMK Runs on 16 datasets, Measured by ACC.

λ	λ = 0	λ = 0.25	λ = 0.5	λ = 0.75	λ = 1
Dataset					
Breast-cancer	0.6032	0.6032	0.6471	0.6471	**0.6471**
Shuttle	0.4598	0.4598	0.4598	0.5994	**0.8327**
Pendigits	**0.7424**	0.6755	0.6575	0.6161	0.5780
Colon-cancer	**0.8710**	0.8710	0.8710	0.5484	0.6129
Zoo	0.6436	0.6436	0.6436	0.7624	**0.8416**
Haberman	0.5000	0.5000	0.5000	0.7582	**0.7582**
Svmguide2	0.4501	0.4501	0.4501	0.5985	**0.5985**
Wine	**0.7022**	0.7022	0.7022	0.7022	0.5730
Ionosphere	**0.7123**	0.7123	0.7123	0.6439	0.6439
Leukemia	0.5882	0.5882	0.5882	0.6176	**0.6176**
Gisette	0.6650	0.6650	0.6700	0.6700	**0.6700**
Splice	0.5160	0.6560	0.6560	0.6560	**0.6560**
Svmguide4	**0.2967**	0.2967	0.2600	0.2933	0.2633
Liver-disorders	**0.7448**	0.7448	0.7448	0.7103	0.7103
Soybean-small	**1**	1	1	0.7234	0.7447
Balance-scale	0.5488	0.6016	0.6016	0.5264	**0.6144**

The optimal results for the corresponding dataset are denoted in bold.

**TABLE 4:** AIMK Runs on 16 datasets, Measured by RI.

λ Dataset	λ = 0	λ = 0.25	λ = 0.5	λ = 0.75	λ = 1
Breast-cancer	0.5206	0.5206	0.5426	0.5426	**0.5426**
Shuttle	0.5600	0.5600	0.5600	0.5799	**0.7578**
Pendigits	**0.9214**	0.9102	0.9074	0.9001	0.8852
Colon-cancer	**0.7715**	0.7715	0.7715	0.4966	0.5177
Zoo	0.7580	0.7580	0.7580	0.9115	**0.9228**
Haberman	0.4984	0.4984	0.4984	0.6321	**0.6321**
Svmguide2	**0.5669**	0.5669	0.5669	0.5622	0.5622
Wine	**0.7187**	0.7187	0.7187	0.7187	0.6919
Ionosphere	**0.5889**	0.5889	0.5889	0.5401	0.5401
Leukemia	0.5009	0.5009	0.5009	0.5134	**0.5134**
Gisette	0.5540	0.5540	0.5574	0.5574	**0.5574**
Splice	0.5000	0.5482	0.5482	0.5482	**0.5482**
Svmguide4	**0.7219**	0.7219	0.6999	0.7205	0.6698
Liver-disorders	**0.6172**	0.6172	0.6172	0.5856	0.5856
Soybean-small	**1**	1	1	0.8316	0.8335
Balance-scale	0.5741	0.6724	0.6724	0.5959	**0.6866**

The optimal results for the corresponding dataset are denoted in bold.

**TABLE 5:** AIMK Runs on 16 datasets, Measured by F-Measure.

λ Dataset	λ = 0	λ = 0.25	λ = 0.5	λ = 0.75	λ = 1
Breast-cancer	0.5852	0.5852	0.7027	0.7027	**0.7027**
Shuttle	0.5016	0.5016	0.5016	0.5600	**0.8430**
Pendigits	**0.6180**	0.5966	0.5902	0.5705	0.5317
Colon-cancer	**0.7803**	0.7803	0.7803	0.5118	0.6584
Zoo	0.5999	0.5999	0.5999	0.8051	**0.8297**
Haberman	0.5482	0.5482	0.5482	0.7290	**0.7290**
Svmguide2	0.4283	0.4283	0.4283	0.5255	**0.5255**
Wine	0.5835	0.5835	0.5835	0.5835	**0.5956**
Ionosphere	0.6049	0.6049	0.6049	0.6999	**0.6999**
Leukemia	0.5156	0.5156	0.5156	0.6625	**0.6625**
Gisette	0.5788	0.5788	0.6062	0.6062	**0.6062**
Splice	**0.6662**	0.5551	0.5551	0.5545	0.5545
Svmguide4	0.2029	0.2029	0.2060	0.2000	**0.2178**
Liver-disorders	**0.6798**	0.6798	0.6798	0.6754	0.6754
Soybean-small	**1**	1	1	0.6566	0.6617
Balance-scale	0.4601	0.5721	0.5721	0.4719	**0.5901**

The optimal results for the corresponding dataset are denoted in bold.

### Impact of Threshold *Thr*

To explain more clearly how to use skeleton points to determine the threshold *Thr*, we perform experiments on four representative datasets: Pendigits, Shuttle, Wine, and Gisette, whose data size and dimensions are from small to large and low to high, respectively. We run AIMK when *Thr* is set as the mean value of the maximum weights, mean weights, and minimum weights of adjacent edges of each skeleton point. Meanwhile, λ is set as 0 or 1, and the final results are shown as both sides of the slash “/’’, respectively. We use ACC to evaluate the results of each run. The results are shown in Table 6. For each dataset, the optimal results can be obtained only when *Thr* is set as the maximum weights of adjacent edges of each skeleton point. Therefore, it is more reasonable to set *Thr* as the maximum weights of adjacent edges of each skeleton point. Furthermore, the threshold *Thr* can be also used to help density-based clustering algorithms, such as DBSCAN (Ester et al., 1996), OPTICS (Ankerst et al., 1999), and SFDP, calculate the density of points without any extra adjusting parameters.

**TABLE 6:** The impact of Threshold *Thr* on clustering performance.

*Thr* Dataset	Min	Mean	Max
Pendigits	0.5780/0.5780	0.5780/.5780	**0.7424**/.5780
Shuttle	0.7530/.7530	0.7530/.7530	0.4598/.8327
Wine	0.5730/.5730	0.5730/.5730	**0.7022**/.5730
Gisette	0.5010/.5010	0.5010/.5010	0.6650/.6700

The optimal results for the corresponding dataset are denoted in bold.

### Comparison With Other Clustering Algorithms

We compare the clustering performance between AIMK (λ is set as 0 or 1) and 11 selected algorithms by 16 normal real-world datasets. ACC, RI, and F-measure are exploited to evaluate the performance of each baseline on each dataset. The results are listed in Tables 7–9. The optimal results for the corresponding dataset are denoted in bold. We use the average rank to measure the final performance of each baseline across datasets. The rank means the rank number of each row sorted in descending order. If there are the same results from two different algorithms, their ranks are equal.

**TABLE 7:** Results of All Algorithms on 16 Real-World datasets, Measured by ACC.

Algorithm	K-means	K-means++	KT	MSTI	HD	K-medoids	SFDP	FCM	SH	SS	FINCH	AIMK (λ = 0)	AIMK (λ = 1)
Dataset													
Breast-cancer	0.6032	0.6252	0.6471	0.6032	**0.6471**	**0.6471**	0.5928	0.6032	**0.6471**	**0.6471**	**0.6471**	0.6032	**0.6471**	
Shuttle	0.4384	0.4588	0.6590	0.5858	**0.8327**	0.4683	0.4130	0.4002	0.7914	0.3386	0.3477	0.4598	**0.8327**
Pendigits	0.6479	0.6609	0.5895	0.6795	0.5780	0.6553	0.6832	0.6048	0.1123	0.6685	0.6744	**0.7424**	0.5780
Colon-cancer	0.5613	0.6194	0.7742	0.5161	0.6129	0.6258	0.6818	0.5758	0.6290	0.5226	0.5000	**0.8710**	0.6129
Zoo	0.6644	0.7188	0.7327	0.7921	**0.8416**	0.7921	0.5644	0.5752	0.6238	0.5406	0.7921	0.6436	**0.8416**
Haberman	0.5408	0.5121	0.5000	0.5196	0.5196	0.5196	0.5698	0.5098	0.7386	0.5196	0.5163	0.5000	**0.7582**
Svmguide2	0.4624	0.4737	0.4680	0.5115	0.4655	0.4680	0.4076	0.5151	0.5703	0.4760	**0.5985**	0.4501	**0.5985**
Wine	0.6893	0.6640	0.5730	0.7022	0.7022	0.6820	**0.7079**	0.6854	0.3764	**0.7079**	0.6124	0.7022	0.5730
Ionosphere	0.7103	0.7100	0.7094	**0.7123**	0.7094	0.7094	0.5335	0.7094	0.6439	**0.7123**	0.5413	**0.7123**	0.6439
Leukemia	0.5765	0.5882	0.5588	0.5294	0.5882	0.5294	0.5294	0.5294	**0.6176**	0.5588	0.5000	0.5882	**0.6176**
Gisette	0.6538	0.6548	0.6540	0.6690	0.6650	0.6281	0.6300	0.6595	0.5010	0.6664	0.5540	0.6650	**0.6700**
Splice	0.6409	0.6539	0.6550	0.6540	0.6550	0.5990	0.5070	0.6283	0.5160	0.6476	0.5220	0.5160	**0.6560**
Svmguide4	0.2720	0.2597	0.2633	0.2633	0.2867	0.2620	0.2500	0.2590	0.1967	0.2653	**0.3067**	0.2967	0.2633
Liver-disorders	0.7283	0.7269	0.7103	0.7103	0.7103	0.7034	0.6038	0.7241	0.6276	0.6745	0.6966	**0.7448**	0.7103
Soybean-small	0.7191	0.7319	0.7447	0.7660	0.7447	0.8085	0.8936	0.7234	**1**	0.7787	0.8936	**1**	0.7447
Balance-scale	0.5144	0.5179	0.4400	0.5408	**0.6144**	0.5363	0.5439	0.5245	0.4640	0.4104	0.4608	0.5488	**0.6144**
Rank	7.062	6.750	6.562	5.062	4.438	6.375	7.688	7.875	7.062	6.312	7.375	1.188	

The optimal results for the corresponding dataset are denoted in bold.

According to Tables 7–9, AIMK (set λ as 0 or 1) achieves the best performance on 14, 13, and 8 of the 16 datasets when measured by ACC, RI, and F-measure, respectively. Moreover, it can be seen from the ranks that AIMK is obviously superior to the other 11 baselines, no matter which validation index we use.

Furthermore, according to Table 7, AIMK achieves the highest ACC rank compared with the other 11 baselines. The rank of AIMK 1.188 is much higher than the rank of HD 4.438, which achieves the second-highest ACC rank. FCM achieves the lowest ACC rank, at just 7.875. HD is the best-performing initialization method for K-means in addition to AIMK in Table 7, whose rank is 4.438. According to Table 8, AIMK achieves the highest RI rank compared with the other 11 baselines. The rank of AIMK 1.312 is much higher than the rank of HD 4.438, which achieves the second-highest RI rank. FINCH achieves the lowest RI, at just 9.000. HD is still the best-performing initialization method for K-means in addition to AIMK in Table 8, whose rank is 4.438. According to Table 9, AIMK still achieves the highest F-measure rank compared with the other 11 baselines. The rank of AIMK 1.938 is higher than the rank of SH 3.125, which achieves the second-highest F-measure rank. FCM achieves the lowest F-measure, which is just 10.00. MSTI is the best-performing initialization method for K-means in addition to AIMK in Table 9, whose rank is 5.938.

**TABLE 8:** Results of All Algorithms on 16 Real-World datasets, Measured by RI.

Algorithm	K-means	K-means++	KT	MSTI	HD	K-medoids	SFDP	FCM	SH	SS	FINCH	AIMK (λ = 0)	AIMK (λ = 1)
Dataset													
Breast-cancer	0.5228	0.5338	**0.5426**	0.5206	**0.5426**	**0.5426**	0.5179	0.5206	**0.5426**	**0.5426**	**0.5426**	0.5206	**0.5426**
Shuttle	0.5201	0.5567	0.5846	0.5802	**0.7578**	0.5652	0.4847	0.5115	0.6520	0.4735	0.4023	0.5600	**0.7578**
Pendigits	0.9021	0.9148	0.8963	0.9079	0.8852	0.9098	0.9193	0.8869	0.1147	0.9165	0.9083	**0.9214**	0.8852
Colon-cancer	0.5101	0.5334	0.6446	0.4923	0.5177	0.5454	0.5618	0.5015	0.5256	0.4961	0.4918	**0.7715**	0.5177
Zoo	0.8283	0.8786	0.8618	0.8994	**0.9228**	0.8953	0.7657	0.8386	0.7186	0.8088	0.8994	0.7580	**0.9228**
Haberman	0.4989	0.5122	0.4984	0.4991	0.4991	0.4991	0.5081	0.4986	0.6126	0.4991	0.4989	0.4984	**0.6321**
Svmguide2	0.5621	0.5646	0.5544	0.5738	0.5532	**0.5812**	0.4905	0.5585	0.4317	0.5610	0.5158	0.5669	0.5622
Wine	0.7079	0.7049	0.6919	0.7187	0.7187	0.7172	0.7191	0.7105	0.3479	**0.7204**	0.6262	0.7187	0.6919
Ionosphere	0.5880	0.5870	0.5865	**0.5889**	0.5865	0.5865	0.5054	0.5865	0.5401	**0.5889**	0.5020	**0.5889**	0.5401
Leukemia	0.4955	0.4898	0.4920	0.4866	0.5009	0.4866	0.4866	0.4866	**0.5134**	0.4920	0.4848	0.5009	**0.5134**
Gisette	0.5481	0.5534	0.5470	0.5567	0.5540	0.5298	0.5333	**0.5610**	0.4995	0.5549	0.5053	0.5540	0.5574
Splice	0.5467	0.5471	0.5476	0.5470	0.5476	0.5191	0.4996	0.5262	0.5000	0.5434	0.5005	0.5000	**0.5482**
Svmguide4	0.7078	0.7010	0.6906	0.6702	0.7181	0.6895	0.7178	0.7208	0.1885	0.7159	0.6337	**0.7219**	0.6698
Liver-disorders	0.6064	0.5932	0.5856	0.5856	0.5856	0.5799	0.5186	0.5977	0.5293	0.5560	0.5743	**0.6172**	0.5856
Soybean-small	0.8286	0.8313	0.8335	0.8372	0.8335	0.8501	0.8982	0.8316	**1**	0.8417	0.8982	**1**	0.8335
Balance-scale	0.5852	0.5888	0.5428	0.6171	0.6866	0.5889	0.5801	0.6008	0.4329	0.5354	0.4299	0.5741	0.6866
Rank	7.000	6.000	6.500	5.500	4.438	5.812	7.625	7.062	8.062	5.938	9.000	1.312	

The optimal results for the corresponding dataset are denoted in bold.

**TABLE 9:** Results of All Algorithms on 16 Real-World datasets, Measured by F-Measure.

Algorithm	K-means	K-means++	KT	MSTI	HD	K-medoids	SFDP	FCM	SH	SS	FINCH	AIMK (λ = 0)	AIMK (λ = 1)
Dataset													
Breast-cancer	0.5969	0.6557	**0.7027**	0.5852	**0.7027**	**0.7027**	0.5922	0.5852	**0.7027**	**0.7027**	**0.7027**	0.5852	**0.7027**
Shuttle	0.4291	0.4966	0.5909	0.5603	**0.8430**	0.5149	0.4199	0.4134	0.7892	0.3535	0.3179	0.5016	**0.8430**
Pendigits	0.5679	0.5997	0.5499	0.5933	0.5316	0.5809	0.6267	0.5264	0.1816	0.5912	**0.6280**	0.6180	0.5317
Colon-cancer	0.5339	0.5625	0.6582	0.5176	0.6584	0.5587	0.6610	0.5151	0.6843	0.5096	0.6037	**0.7803**	0.6584
Zoo	0.6227	0.7171	0.6984	0.7608	**0.8297**	0.7536	0.4601	0.5845	0.6169	0.5435	0.7608	0.5999	**0.8297**
Haberman	0.5492	0.5669	0.5482	0.5504	0.5480	0.5479	0.5945	0.5479	**0.7583**	0.5480	0.5494	0.5482	0.7290
Svmguide2	0.4238	0.4274	0.4143	0.4436	0.4125	0.4487	0.4289	0.4543	0.5986	0.4215	**0.6260**	0.4283	0.5255
Wine	0.5883	0.5885	0.5956	0.5835	0.5835	0.5858	0.5834	0.5728	0.4959	0.5859	**0.5962**	0.5835	0.5956
Ionosphere	0.6041	0.6032	0.6028	0.6049	0.6028	0.6024	0.5929	0.6028	**0.6999**	0.6041	0.6285	0.6049	**0.6999**
Leu	0.5240	0.5530	0.4991	0.5727	0.5156	0.5017	0.4875	0.4875	**0.6625**	0.4991	0.6142	0.5156	**0.6625**
Gisette	0.5772	0.5895	0.5860	0.6053	0.5788	0.6041	0.6157	0.5640	**0.6658**	0.5583	0.6442	0.5788	0.6062
Splice	0.5533	0.5538	0.5540	0.5539	0.5543	0.5215	0.5750	0.5272	**0.6662**	0.5478	0.6639	**0.6662**	0.5545
Svmguide4	0.1990	0.2012	0.2087	0.2171	0.1975	0.2064	0.1829	0.1908	**0.2835**	0.1979	0.2415	0.2029	0.2178
Liver-disorders	0.6753	0.6739	0.6654	0.6754	0.6754	0.6683	0.5815	0.6567	**0.6887**	0.6001	0.6821	0.6798	0.6754
Soybean-small	0.6745	0.6882	0.6617	0.6716	0.6617	0.6955	0.7925	0.6566	**1**	0.7080	0.8173	**1**	0.6617
Balance-scale	0.4578	0.4629	0.4028	0.4991	0.5901	0.4724	0.4983	0.4827	**0.6016**	0.3919	0.6013	0.4601	0.5901
Rank	7.812	6.562	7.062	5.938	6.375	7.188	7.500	10.00	3.125	8.688	3.188	1.938	

The optimal results for the corresponding dataset are denoted in bold.

**FIGURE 2:**
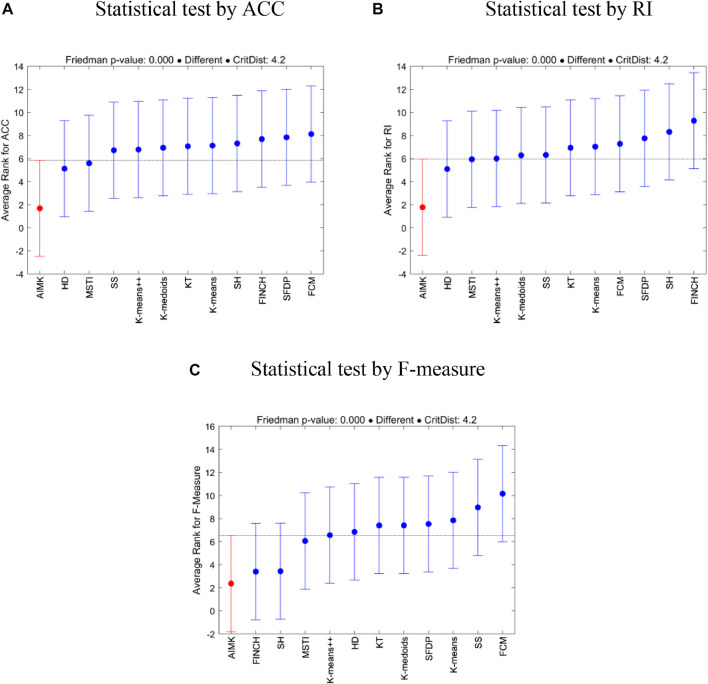
MCB test for AIMK and the compared baselines.

To further investigate the statistical differences between the compared baselines and AIMK, we employ multiple comparisons with the best (MCB) test (Koning et al., 2005). The test computes the average ranks (in error rates, that is, 1-index values) of the forecasting methods according to the specific metric across all datasets of the competition and concludes whether or not these are statistically different. Figure 2 presents the results of the analysis. In addition, the Friedman *p*-values under the three indices (ACC, RI, and F-measure) are $1.48\times 10^{-5}$ , $1.1\times 10^{-6}$ and $1.1\times 10^{-11}$ , respectively. Therefore, in a big picture, we can conclude AIMK provides significantly better performance than the other 11 compared baselines.

### Performance of AIMK-RS

AIMK-RS is compared with two widely used initialization methods, K-means and K-means++, whose time complexity is also O(n) , on six larger or higher-dimensional datasets. ACC, RI, and F-measure are also exploited to evaluate the results. In addition, we take the average performance of 100 runs as the real performance of the AIMK-RS because it provides for more even sampling and can fully express the characteristics of the original datasets. The optimal results for the corresponding datasets are denoted in bold. The results are listed in Tables 10–12, and we can conclude that compared with the two baselines, AIMK-RS still achieves better performance. Particularly, on the dataset Ijcnn1, ACC, RI, and F-measure of AIMK-RS are increased by 7.68, 7.32, and 6.59%, respectively.

**TABLE 10:** Larger or Higher-Dimensional datasets, Measured by ACC.

Algorithm	K-means	K-means++	AIMK-RS (λ = 0)	AIMK-RS (λ = 1)
Dataset				
Ijcnn1	0.7332	0.7472	0.6712	**0.8240**
Phishing	0.5696	0.5715	**0.6208**	0.5260
Protein	0.4252	0.4258	0.4173	**0.4576**
Mushrooms	0.7918	0.8083	0.8027	**0.8241**
SensIT Vehicle (seismic)	0.4546	0.4657	0.4425	**0.4855**
SensIT Vehicle (combined)	0.5576	0.5598	**0.5636**	0.5384

The optimal results for the corresponding dataset are denoted in bold.

**TABLE 11:** Larger or Higher-Dimensional datasets, Measured by RI.

Algorithm	K-means	K-means++	AIMK-RS (λ = 0)	AIMK-RS (λ = 1)
Dataset				
Ijcnn1	0.6207	0.6367	0.5603	**0.7099**
Phishing	0.5309	0.5315	**0.5682**	0.5020
Protein	0.4390	0.4307	**0.4476**	0.3666
Mushrooms	0.6989	0.7206	0.7126	**0.7300**
SensIT Vehicle (seismic)	0.5650	0.5653	**0.5658**	0.5517
SensIT Vehicle (combined)	0.5941	0.5965	**0.5975**	0.5709

The optimal results for the corresponding dataset are denoted in bold.

**TABLE 12:** Larger or Higher-Dimensional datasets, Measured by F-Measure.

Algorithm	K-means	K-means++	AIMK-RS (λ = 0)	AIMK-RS (λ = 1)
Dataset				
Ijcnn1	0.7439	0.7581	0.6896	**0.8240**
Phishing	0.5933	0.5937	**0.6046**	0.5733
Protein	0.4780	0.4825	0.4683	**0.5268**
Mushrooms	0.7253	0.7428	0.7364	**0.7595**
SensIT Vehicle (seismic)	0.4041	0.4118	0.3949	**0.4374**
SensIT Vehicle (combined)	0.4506	0.4513	0.4495	**0.4627**

The optimal results for the corresponding dataset are denoted in bold.

### Initialize Other Clustering Algorithms Using AIMK

In some variants of the K-means algorithm, the initialization method still plays an important role in the final clustering performance. For example, the initialization is required in the first step of the K-medoids algorithm and the last step of spectral clustering. However, the random initialization method is applied in the original algorithms. In this section, we even leverage AIMK to initialize the K-medoids clustering algorithm and spectral clustering algorithm. Due to the instability of original K-medoids and spectral clustering, we take the average performance of 10 runs as their real performance. There is a hyperparameter δ in the spectral clustering algorithm, so we use the self-tuning mode (Zelnik-Manor and Perona, 2005). The results are shown in Tables 13 and 14. In Table 13, the clustering performance of K-medoids initialized by AIMK, denoted as K-medoids + AIMK reaches the higher or equal performance on 15 datasets, except for the Gisette one. Particularly, the performance is increased by 36.44 and 8.39% on the Shuttle and Colon-cancer datasets, respectively. In Table 14, the overall higher clustering performance can be explored through spectral clustering initialized by AIMK, denoted as Spectral + AIMK. Especially Spectral + AIMK leads to 6.91 and 14.48% higher accuracy on the dataset Pendigits, Balance-scale, respectively.

**TABLE 13:** Use AIMK to initialize the K-Medoids, measured by ACC.

Algorithm	K-medoids	K-mediods + AIMK (λ = 0)	K-medoids + AIMK (λ = 1)
Dataset			
Breast-cancer	0.6471	**0.6471**	**0.6471**
Shuttle	0.4683	0.4280	**0.8327**
Pendigits	0.6553	**0.6830**	0.5732
Colon-cancer	0.6258	0.5000	**0.7097**
Zoo	0.7921	**0.7921**	**0.7921**
Haberman	0.5196	**0.5196**	**0.5196**
Svmguide2	0.4680	**0.4680**	**0.4680**
Wine	0.6820	**0.7079**	**0.7079**
Ionosphere	0.7094	**0.7094**	**0.7094**
Leukemia	0.5294	**0.5294**	**0.5294**
Gisette	**0.6281**	0.6230	0.6230
Splice	0.5990	**0.5990**	**0.5990**
Svmguide4	0.2620	0.2567	**0.2667**
Liver-disorders	0.7034	**0.7034**	**0.7034**
Soybean-small	0.8085	**0.8085**	**0.8085**
Balance-scale	0.5363	0.5136	**0.5648**

The optimal results for the corresponding dataset are denoted in bold.

**TABLE 14:** Use AIMK to initialize spectral clustering, measured by ACC.

Algorithm	Spectral	Spectral + AIMK (λ = 0)	Spectral + AIMK (λ = 1)
Dataset			
Breast-cancer	0.6471	**0.6471**	**0.6471**
Shuttle	0.3386	0.3135	**0.3561**
Pendigits	0.6685	0.6898	**0.7376**
Colon-cancer	0.5226	**0.5484**	**0.5484**
Zoo	0.5406	**0.5446**	0.5050
Haberman	0.5196	**0.5196**	**0.5196**
Svmguide2	0.4760	0.4731	**0.4936**
Wine	0.7079	**0.7079**	**0.7079**
Ionosphere	0.7123	**0.7123**	**0.7123**
Leukemia	0.5588	**0.5588**	**0.5588**
Gisette	0.6664	**0.6670**	0.6650
Splice	0.6476	**0.6498**	0.6440
Svmguide4	0.2653	0.2567	**0.2700**
Liver-disorders	0.6745	0.6690	**0.6828**
Soybean-small	**0.7787**	0.7660	0.7447
Balance-scale	0.4104	**0.5552**	0.5296

The optimal results for the corresponding dataset are denoted in bold.

## Discussion

### Choice of λ

**FIGURE 3:**
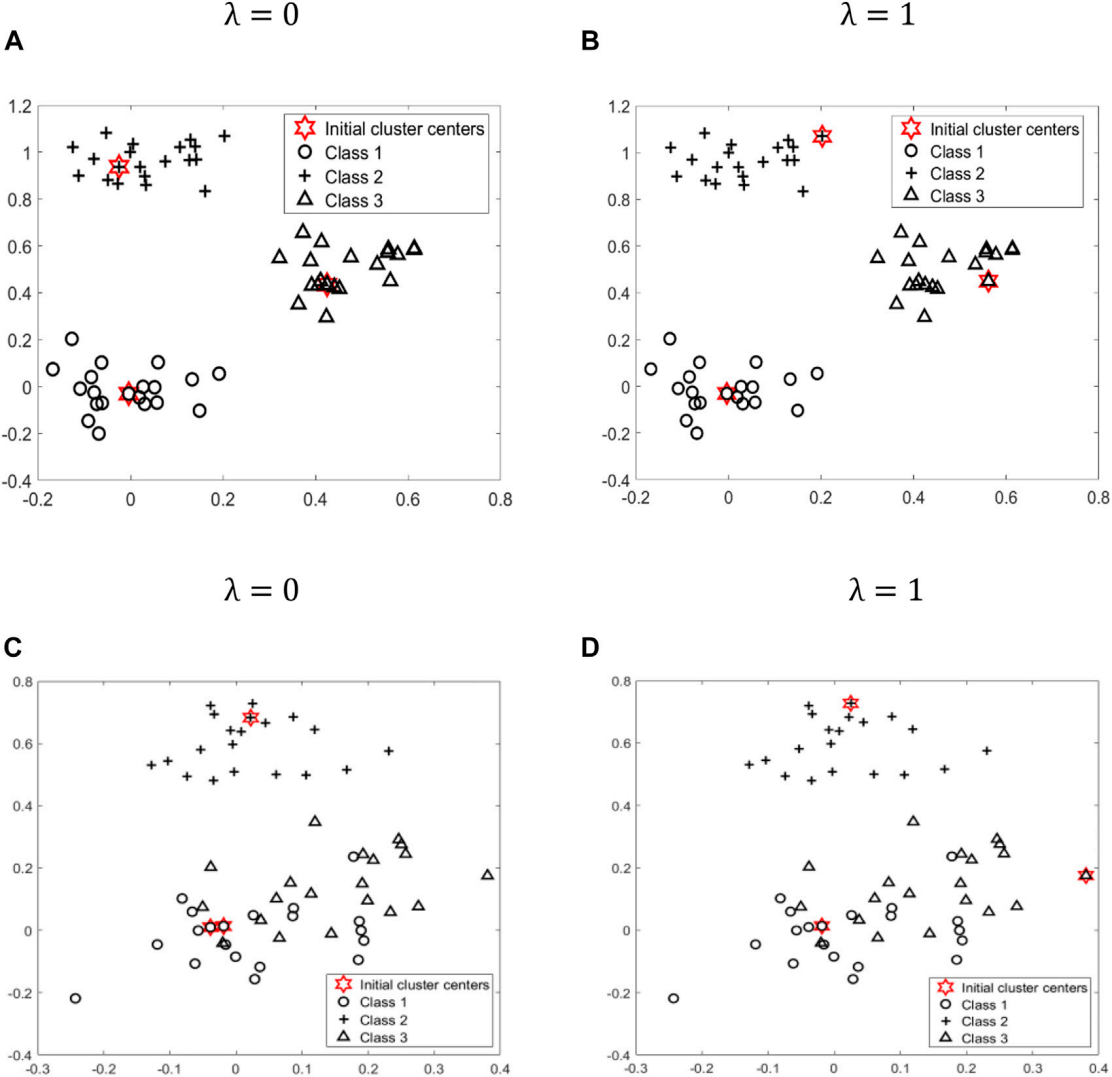
To further illustrate the impact of parameter λ, we generate two types of datasets, **(A)**, **(B)** and **(C)**, **(D)**, with different distribution from a mixture of three bivariate Gaussian densities. Class 1, Class 2, Class 3, and initial cluster centers are represented by different shapes: circle, cross, triangle, and star, respectively.

After the above experiments, we can see that the parameter λ is crucial for the final clustering results. To further illustrate the impact of parameter λ, we generate two types of datasets with different distributions from a mixture of three bivariate Gaussian densities. Figure 3A, Figure 3B is given by

$$\frac{1}{3}Gaussian\left(\begin{array}{c} 0\\ 0 \end{array}\right)\left(\begin{array}{cc} 0.01 & 0\\ 0 & 0.01 \end{array}\right)+\frac{1}{3}Gaussian\left(\begin{array}{c} 0\\ 1 \end{array}\right)\left(\begin{array}{cc} 0.01 & 0\\ 0 & 0.01 \end{array}\right)+\frac{1}{3}Gaussian\left(\begin{array}{c} 0.5\\ 0.5 \end{array}\right)\left(\begin{array}{cc} 0.01 & 0\\ 0 & 0.01 \end{array}\right)$$

Figures 3C,D is given by $\frac{1}{3}Gaussian\left(\begin{array}{c} 0\\ 0 \end{array}\right)\left(\begin{array}{cc} 0.01 & 0\\ 0 & 0.01 \end{array}\right)+\frac{1}{3}Gaussian\left(\begin{array}{c} 0\\ 0.6 \end{array}\right)\left(\begin{array}{cc} 0.01 & 0\\ 0 & 0.01 \end{array}\right)+\frac{1}{3}Gaussian\left(\begin{array}{c} 0.15\\ 0.15 \end{array}\right)\left(\begin{array}{cc} 0.01 & 0\\ 0 & 0.01 \end{array}\right)$ . where Gaussian (X, Y) is a Gaussian normal distribution with the mean X and the covariance matrix Y. We stimulate three clusters, namely, Class 1, Class 2 and Class 3, which are represented by different shapes: circle (20 points), cross (20 points), and triangle (20 points), respectively. As shown in Figures 3A–D, we use AIMK to determine the initial cluster centers marked with the star when λ is set as 0 and 1. In Figure 3A, Figure 3B, when λ is equal to 0, the three cluster centers happen to be the centroid of three classes. When λ is equal to 1, only one cluster center is the centroid of class 1, and the other two cluster centers are just outliers in class 2 and class 3, respectively. In Figure 3C, Figure 3D, when λ is equal to 0, two cluster centers are dropped in class 1, one cluster center is dropped in class 2, and no cluster center is dropped in class 3. However, when λ is equal to 1, three cluster centers happen to be dropped in three classes, and two of the three are outliers.

According to formula (Eq. 4), when λ is equal to 0, only the top K points with a higher density are selected as initial cluster centers. At this time, if all or most of these K initial cluster centers fall in K different classes, as shown in Figure 3A, then the initialization effect is better. However, for some datasets, such as overlapping datasets, shown as Figure 3C, the top K points with higher density cannot be distributed relatively evenly among K classes. Therefore, at this time, we need to consider the distance factor. According to formula (Eq. 4), when λ is equal to 1, we only select the K points that are far apart from each other as initial cluster centers. At this time, all or most of these K initial cluster centers are more likely to be relatively evenly distributed among the K classes, as shown in Figure 3D.

In summary, if the users have prior knowledge of the datasets, the parameter λ can be selected more accurately like the examples above in advance. Otherwise, they can still get good clustering results by executing the algorithm in just two interactions.

## Conclusion

In this study, we propose the AIMK algorithm which can not only adapt to datasets with various characteristics but also obtain better clustering results within two runs. First, we propose a new threshold to calculate the density of the data points based on the skeleton points of MST. Second, we compute the hybrid distances based on the density of the data points. Finally, we select K data points, where the hybrid distances among them are relatively large, as the final cluster centers. In addition, we apply random sampling to AIMK to obtain the AIMK-RS, whose time complexity is only O(*n*).

In the experiment part, first, we analyze the sensitivity of parameter λ on each dataset, and conclude that better performance can be obtained when λ is 0 or 1. Second, we compare AIMK with 11 different algorithms on 16 normal datasets. The experimental results show that AIMK outperforms the current initialization methods and several well-known clustering algorithms. We also compare AIMK-RS with two widely used initialization methods with linear complexity. AIMK-RS still achieves better performance. Particularly, the ACC, RI, and F-measure are increased by 7.68, 7.32, and 6.59% on the dataset Ijcnn1, respectively. Finally, we exploit AIMK to initialize the variants of K-means, such as K-medoids and spectral clustering. The better clustering performance demonstrates AIMK is a good way for initialization and has the potential to extend to other state-of-the-art algorithms. Particularly, for the K-medoids initialized by AIMK, the performance is increased by 36.44 and 8.39% on the Shuttle and Colon-cancer datasets, respectively. Similarly, spectral clustering initialized by AIMK leads to 6.91 and 14.48% higher accuracy on the dataset Pendigits, Balance-scale, respectively. In the discussion part, we take two toy examples to show the choice of λ for datasets with different characteristics.

In the future, we will combine AIMK or AIMK-RS with other state-of-art algorithms to more real-world datasets. Moreover, we will leverage them to some specific applications, such as image segmentation, classification of EEG data, etc.

## Data Availability

The datasets generated for this study are available on request to the corresponding author or Jie Yang (jie.yang-4@student.uts.edu.au).
